# The Tennessee Medicaid medication therapy management program: early stage contextual factors and implementation outcomes

**DOI:** 10.1186/s12913-021-07193-7

**Published:** 2021-11-02

**Authors:** Kenneth C. Hohmeier, Chelsea Renfro, Kea Turner, Parin Patel, Estrella Ndrianasy, Renee Williams-Clark, Lora Underwood, Justin Gatwood

**Affiliations:** 1 Department of Clinical Pharmacy and Translational Science, Memphis, USA; 2 College of Pharmacy, University of Tennessee Health Science Center, Memphis, USA; 3 Department of Health Outcomes and Behavior, Moffitt Cancer Center, Tampa, USA; 4 Department of Oncological Sciences, University of South Florida, Tampa, USA; 5 TennCare, Nashville, USA

**Keywords:** Implementation science, CFIR, Medication therapy management, MTM, Community pharmacy, Community pharmacist, Patient centered medical home, Medicaid

## Abstract

**Background:**

First investigated in the 1990s, medication therapy management (MTM) is an evidence-based practice offered by pharmacists to ensure a patient’s medication regimen is individualized to include the safest and most effective medications. MTM has been shown to a) improve quality of patient care, b) reduces health care costs, and c) lead to fewer medication-related adverse effects. However, there has been limited testing of evidence-based, a-priori implementation strategies that support MTM implementation on a large scale.

**Methods:**

The study has two objectives assessed at the organizational and individual level: 1) to determine the adoption, feasibility, acceptability and appropriateness of a multi-faceted implementation strategy to support the MTM pilot program in Tennessee; and 2) to report on the contextual factors associated with program implementation based on the Consolidated Framework for Implementation Research (CFIR). The overall design of the study was a hybrid type 2 effectiveness-implementation study reporting outcomes of Tennessee state Medicaid’s (TennCare) MTM Pilot program. This paper presents early stage implementation outcomes (e.g., adoption, feasibility, acceptability, appropriateness) and explores implementation barriers and facilitators using the CFIR. The study was assessed at the (a) organizational and (b) individual level. A mixed-methods approach was used including surveys, claims data, and semi-structured interviews. Interview data underwent initial, rapid qualitative analysis to provide real time feedback to TennCare leadership on project barriers and facilitators.

**Results:**

The total reach of the program from July 2018 through June 2020 was 2033 MTM sessions provided by 17 Medicaid credentialed pharmacists. Preliminary findings suggest participants agreed that MTM was acceptable (μ = 16.22, SD = 0.28), appropriate (μ = 15.33, SD = 0.03), and feasible (μ = 14.72, SD = 0.46). Each of the scales had an excellent level of internal (> 0.70) consistency (feasibility, α = 0.91; acceptability, α = 0.96; appropriateness, α = 0.98;). Eight program participants were interviewed and were mapped to the following CFIR constructs: *Process, Characteristics of Individuals, Intervention Characteristics, and Inner Setting.* Rapid data analysis of the contextual inquiry allowed TennCare to alter initial implementation strategies during project rollout.

**Conclusion:**

The early stage implementation of a multi-faceted implementation strategy to support delivery of Tennessee Medicaid’s MTM program was found to be well accepted and appropriate across multiple stakeholders including providers, administrators, and pharmacists. However, as the early stage of implementation progressed, barriers related to relative priority, characteristics of the intervention (e.g., complexity), and workflow impeded adoption. Programmatic changes to the MTM Pilot based on early stage contextual analysis and implementation outcomes had a positive impact on adoption.

**Supplementary Information:**

The online version contains supplementary material available at 10.1186/s12913-021-07193-7.

## Background

Medication therapy management (MTM) is a service offered by pharmacists to ensure a patient’s medication regimen is individualized to include the safest and most effective medications [1]. Originating in 1990s, MTM is an evidence-based practice that has grown out of early pilot projects demonstrating the positive impact of pharmacist-provided medication management on clinical outcomes. Specifically, MTM has been shown to a) improve quality of patient care, b) reduces health care costs, and c) lead to fewer medication-related adverse effects [2–4].

Given its effectiveness, MTM is now considered an evidence-based intervention by the Centers for Disease Control and Prevention [5, 6]. As the U.S. healthcare system moves toward a value-based model, payers are placing an increased emphasis on MTM delivery. Most notably, the Centers for Medicare and Medicaid Services (CMS)—the largest payer for MTM—recently expanded MTM access to additional beneficiaries through the Enhanced MTM Model [7, 8]. Additionally, many state Medicaid programs have implemented MTM services to lower expenses while maintaining or enhancing their quality of care [9]. Despite a robust body of research on its effectiveness, there is lack of evidence on MTM implementation.

Implementing MTM with high fidelity is critical to ensure consistent patient outcomes; however, studies suggest there is wide variation in both how MTM is implemented and what elements of MTM are involved in implementation [6, 9]. Like many evidence-based practices, implementing and scaling MTM across settings and locations has proven challenging. Pharmacists have reported numerous barriers to providing MTM services including difficulty forming partnerships with healthcare providers, lack of knowledge about how best to implement MTM services, and lack of reimbursement [10, 11]. Additionally, pharmacists often lack the necessary data to deliver MTM effectively, such as clinical data from health care providers, or quality monitoring systems that allow pharmacists to track the quality of MTM implementation [12].

To overcome barriers to MTM services, some payers have tested implementation strategies, such as creating web-based applications that allow for documentation, integration of clinical data from providers, and tracking of patient outcomes to facilitate better collaboration between physicians and pharmacists [12]. Pharmacies themselves too have tried a variety of implementation strategies with varying success [13]. Additionally, researchers have started to develop frameworks and implementation strategies to help pharmacists adapt MTM for their local setting [14]. However, there has been limited testing of evidence-based, a-priori implementation strategies that support MTM implementation on a large scale.

To address this gap, this study has two objectives assessed at the organizational and individual level: 1) to determine the adoption, feasibility, acceptability and appropriateness of a multi-faceted implementation strategy to support the MTM pilot program in Tennessee; and 2) to report on the contextual factors associated with program implementation based on the Consolidated Framework for Implementation Research (CFIR).

## Methods

### Study setting, intervention, and implementation strategies

Tennessee’s state Medicaid program (TennCare) provides care for 1.4 million Tennesseans. The program primarily serves low-income children, older adults, pregnant women, and individuals with disabilities, roughly 20% of the state’s population. TennCare facilitates managed care for the state’s population by contracting with three managed care organizations.

A current TennCare priority is to improve primary care within the state through new care delivery models that strengthen collaboration across primary care and specialty providers. This Primary Care Transformation (PCT) program aims to improve the quality of primary care services and reducing health care-related costs for Tennessee Medicaid beneficiaries by using a “whole-person” approach to provide seamless access to healthcare. It is comprised of two main elements: (1) Patient-Centered Medical Home (PCMH) and (2) Tennessee Health Link (THL). The latter of which serves as a care coordination program, similar to a PCMH, but specifically for TennCare members with behavioral health needs. TennCare’s role in the PCT program is to support all participating providers through PCT implementation, facilitate team-based care, and provide incentives for meeting or exceeding program requirements. To further support this PCT initiative, in 2018 TennCare launched its MTM Pilot Program to expand collaboration between primary care providers and pharmacists.

The TennCare MTM Pilot program is a multi-faceted implementation strategy that bundles several discrete strategies to integrate the pharmacist into a patient’s care. It was developed by TennCare leadership based on a review of the literature and personal correspondence with experts on barriers and facilitators of other states’ efforts to implement an MTM program for their Medicaid members [9, 15–18]. The multi-faceted implementation strategy includes several discrete approaches that were selected to address known barriers preventing high-quality implementation of MTM. One of the most prominent of these barriers, for example, is forming partnerships between pharmacists and primary care providers [11]. To address this, TennCare MTM pilot required collaborative practice agreements (CPAs) between primary care providers and pharmacists to foster collaboration and permit pharmacists to make medication regimen changes. Another critical barrier to delivering MTM effectively is ensuring adequate compensation of pharmacists for their time. To overcome this barrier, the TennCare MTM pilot developed an alternative payment model to pay pharmacists with rates adjusted based on patient’s risk level (e.g., disease severity, types of medications). Additional strategies and targeted barriers are outlined in Table 1.

**Table 1:** Overview of Multi-faceted Implemented Strategies for the TennCare MTM Pilot Project

Strategy	Description	Target	Timeframe	Barriers addressed
**Planning strategies:** Gathering data, building buy-in, and initiating leadership and partnerships				
Assess readiness and identify barriers and facilitators	University surveys. TennCare and MCO evaluation barriers and challenges of MTM pilot program.	MTM Pharmacists	Early 2019	Limited knowledge of local context
Obtain formal commitments	Formalize CPAs between primary care providers and pharmacists	MCO^a^, Pharmacists	2018–2019	Lack of partnership between pharmacy and primary care
**Education strategies:** Informing stakeholders about intervention implementation				
Conduct ongoing training	TennCare MTM on-site Training Workshop: “You’re an MTM pharmacist, NOW WHAT?”	Pharmacists	Five sessions throughout 2019	Lack of knowledge about high-quality MTM implementation
**Finance strategies:** Incentivize the consistent use of the intervention				
Use alternative payment models^c^	Paying pharmacists using an alternative payment model	Pharmacists	2019–2020	Lack of incentives for MTM
**Restructure strategies:** Creating data systems				
Relay clinical data to providers	Provide data on MTM completion rates	MCO, Pharmacists, Provider, Legislature, CMS^b^	Quarterly 2019–2020	Lack of data systems
**Quality management strategies:** Supporting processes for quality assurance and improvement				
Purposely reexamine implementation	UTHSC Surveys, TennCare Re-evaluation, MCO	MCO, Pharmacists	2019–2020	Lack of quality monitoring systems
Capture and share local knowledge	UTHSC, TennCare use of formal and informal quantitative and qualitative data collection	Pharmacist	2019–2020	

^a^*MCO* Managed care organization

^b^*CMS* Centers for Medicare & Medicaid Services

^c^Value-based payment model ultimately was changed to a fee-for-service model prior to project launch

To engage in the MTM Pilot, pharmacists were required to receive a Tennessee Medicaid identification number, establish a CPA with a PCMH or THL, and complete the credentialing process with at least one of TennCare’s three managed care organizations (MCOs). However, the program was not prescriptive on where pharmacists practiced MTM; consequently, pharmacists primary practice site could include either a traditional community pharmacy or embedded in the PCMH or THL itself.

### Study design

**Fig. 1:**
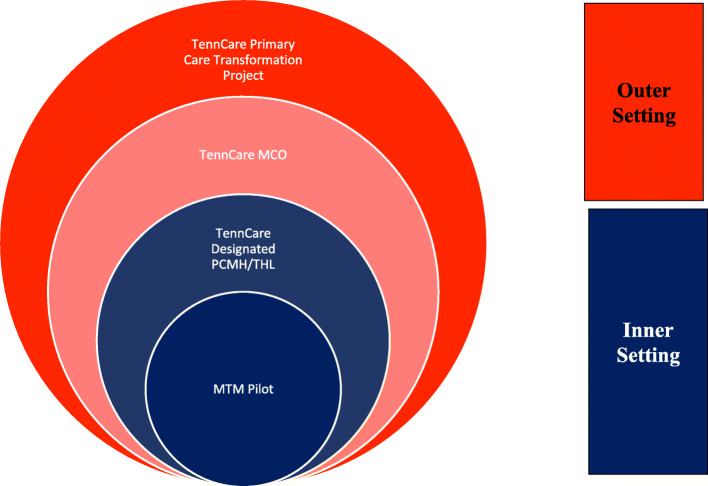
Levels of Implementation Assessment

The overall design of the pilot’s assessment is a hybrid type 2 effectiveness-implementation study. Given the robust effectiveness literature available for MTM, the reporting of a standalone effectiveness trial would contribute little to the overall body knowledge on MTM as an established, evidence based practice; yet, the uniqueness of several aspects of the design of this MTM program (e.g., partnership with PCMHs, use of CPAs, U.S. Medicaid population) effectiveness data is still nonetheless important to assess. In addition, there is a dearth of information in spread and scale of MTM evidence based practices – especially as it relates to implementation barriers and facilitators. Therefore, a hybrid design capturing both implementation and effectiveness outcomes was selected to assess the unique elements of the MTM program and understand how it might be spread and scaled in the future. This paper presents early stage implementation outcomes (e.g., adoption, feasibility, acceptability, appropriateness) and explores implementation barriers and facilitators using the CFIR. The study was assessed at the (a) organizational and (b) individual level (i.e. THL and PCMH) (Fig. 1). A mixed-methods approach was used [19], whereby quantitative and qualitative data were collected simultaneously for the purposes of complementarity (i.e., answering a related set of questions). A rapid analysis approach provided real-time feedback on the program’s implementation to TennCare in order for programmatic changes to be made as needed [20]. The project launched July 1, 2018, after which point credentialed pharmacists were approved to provide reimbursable MTM services. This study was approved by the University of Tennessee Health Science Center Institutional Review Board.

### Study participants

Participants included three main categories of individuals: pharmacists, healthcare administrators, and healthcare providers (e.g., physicians, nurse practitioners, physician assistant). Pharmacists involved in the project generally self-identified into two groups: community pharmacists and ambulatory care pharmacists. Community pharmacists were those in traditional community pharmacy settings who, in addition to clinical responsibilities of direct patient care, were also responsible, to some extent, for medication distribution functions. Ambulatory care pharmacists were those whose primary responsibilities were direct patient care with no medication distribution functions in their primary pharmacy roles and prior to the study were already embedded to some extent in a health-system or medical office setting, as opposed to a brick and mortar community pharmacy.

### Data collection and sources

Surveys were administered to stakeholders participating in the TennCare MTM pilot (*n* = 67). The sampling frame was developed based on an initial list of contact information provided by TennCare for pharmacists, healthcare administrators, and providers (e.g., primary care physicians, nurse practitioners, physician assistants). A modified Dillman approach was used for electronic survey development and dissemination, with four separate email requests sent over a 4-week period of time [21]. A total of 18 surveys were received back, resulting in a 27% response rate. The survey assessed organizational characteristics and participant perceptions about feasibility, acceptability, and appropriateness (described in measures section below).

Eight providers participating in the TennCare MTM pilot were also interviewed. Enrolled pharmacist and PCMH/THL champions (e.g., administrators, medical assistants, schedulers) as of January 2019 were contacted in phases to participate in the interview (*n* = 44). In addition, snowball sampling was also employed by asking for additional suggested informants at the end of each interview. Semi-structured interviews were conducted by a trained qualitative researcher and expert in Medication Therapy Management (KH) and research assistant trained in survey data collection (PP) who had no prior relationships with participants. Interviews were conducted in-person or over the phone at the request of the participant and were transcribed by a third-party transcription service. Field notes were taken by the research assistant and combined with the transcriptions for analysis. Participation was voluntary and anonymous, and no incentive was given for participating.

Billed MTM claims between July 1, 2018 and June 30, 2020 were used to evaluate adoption. Outpatient, inpatient, and pharmacy claims were provided by TennCare for assessing medication adherence, healthcare resource utilization, and costs.

### Measures

Implementation outcomes were based on Proctor’s implementation outcomes framework [22]. Given that this was an early stage implementation assessment, adoption, feasibility, acceptability, and appropriateness were selected as the main outcomes of interest (Table 2). Additionally, a contextual inquiry was performed to elicit factors associated with program implementation [23, 24]. Adoption is represented by claims submissions with approved procedure codes submitted for reimbursement by program pharmacists (CPT: 99605). Initial adoption in this program was indicated by a first MTM session and then subsequent follow-up MTM’s as outlined in the pilot by TennCare and submitted for reimbursement using appropriate codes (CPT:99606, 99,607, 98,966, 98,967, 98,968). Feasibility, acceptability, and appropriateness were measured using the Feasibility Intervention Measure (FIM), the Acceptability of Intervention Measure (AIM), and the Intervention Appropriateness Measure (IAM) [25]. Each measure includes a 4-item scale that contains a 5-item Likert scale ranging from completely disagree (0) to completely agree (4).

**Table 2:** Implementation Measures for Early Implementation Assessment of TennCare’s MTM Pilot Program

Outcome	Individual measures	How measured	When measured
Feasibility Acceptability Appropriateness	• Perceptions about other determinants of implementation (inner setting, process, individual characteristics)	• FIM survey instrument • IAM survey instrument • AIM survey instrument • Interview covering CFIR domains	• Early (pre-implementation to first 3 months)
Adoption	• MTM sessions delivered	• Number of claims submitted to MCO	• Early (first 6 months)

*FIM* Feasibility of Intervention Measure

*IAM* Intervention Appropriateness Measure

*AIM* Acceptability of Implementation Measure

*HEDIS* Healthcare Effectiveness Data and Information Set

*MCO* Managed Care Organization

Contextual inquiry was performed using the CFIR framework using a phenomenological approach [26]. A semi-structured interview guide (Additional file 1) was developed after group deliberations on each construct between University researchers and TennCare leadership until consensus was reached on the final constructs: *structural characteristics, culture, implementation climate, readiness for implementation, knowledge & beliefs about the intervention, planning,* and *engagemen*t. A demographics section was also used to collect participant role, and time at organization. Interviews were audio recorded and transcribed by a third party transcription service. Field notes were recorded on paper and later scanned into data analysis software for analysis. To guide the reporting of qualitative methods and findings, the Consolidated Criteria for Reporting Qualitative studies (COREQ) checklist was used [27].

### Data analysis and dissemination

Interviews continued until a point of saturation [28] and were analyzed using deductive thematic analysis in NVivo. The CFIR framework was used to derive codes and themes from transcriptions and field notes [26]. Codes were further validated by member checking. Emerging themes and results derived from rapid assessment technique [20] were shared quarterly with stakeholders in formal reports and discussed at monthly meetings between the University and TennCare.

Percentages were calculated to describe the study population. Participant responses were summarized on each of the three scales using descriptive statistics. The analyses were conducted using Stata version 16.0 (College Station, TX). There were 20 respondents and five with missing data, which were dropped from the analysis, leaving a total sample size of 15.

## Results

### Early implementation outcomes

#### Adoption and reach

**Fig. 2:**
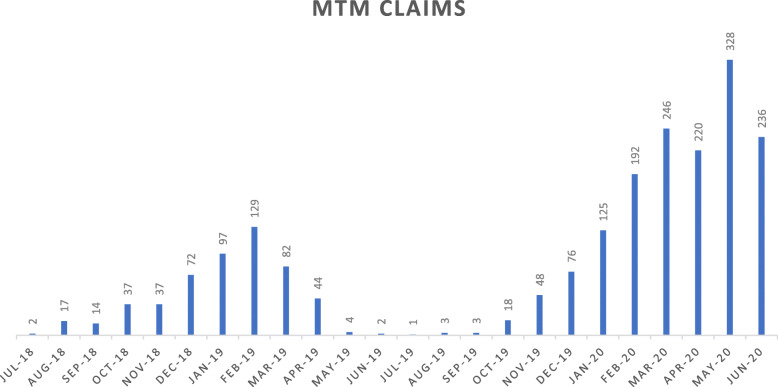
Adoption of TennCare MTM Pilot in Early Stage of Implementation

Adoption of the program included 10 credentialed pharmacist providers through June 2019, which increased to 17 pharmacists by June of 2020. After an initial rise in pharmacist-billed claims from mid-2018 through February of 2019, there was a steep decline in claims into mid-2019 with only 2 MTM claims billed in June 2019 (Fig. 2). This was followed by a sharp increase in MTM claims beginning in June 2019 and continuing through the end of the study period. The total reach of the program through June 30, 2020 was 2033 MTM sessions provided to 1340 patients.

#### Feasibility, acceptability, and appropriateness

Most of the organizations that the survey respondents were affiliated with were either medical offices (40%) or hospitals (33%) (Table 3). The majority (60%) were located in rural areas, had participated in an MTM program in the past (53%), had non-dispensing hours for pharmacists (73%), and had appointed a champion for the pilot program (60%). About a third of pharmacies had a pharmacy technician who could devote time to clinical services (33%).

**Table 3:** Characteristics of Organizations Participating in TennCare MTM Pilot

Characteristics	***N*** = 15
***Organizational type, %***	
Health system	20.0
Hospital	33.3
Medical office	40.0
Community Pharmacy	6.7
***Rural location,*** %	60.0
***MTM participation***, %	53.3
***Non-dispensing hours***, %	73.3
***Pharmacist technician for clinical services,*** %	33.3
***TennCare MTM Champion,*** %	60.0

Preliminary findings suggest participants agreed that MTM was acceptable (μ = 16.22, SD = 0.28), appropriate (μ = 15.33, SD = 0.03), and feasible (μ = 14.72, SD = 0.46) (Table 4). Each of the scales had an excellent level of internal (> 0.70) consistency (feasibility, α = 0.91; acceptability, α = 0.96; appropriateness, α = 0.98;) [29]. On the acceptability scale, the “I like the TennCare MTM Pilot” had the lowest average rating (μ = 3.7, SD = 1.2) and the “I approve of the TennCare MTM Pilot” has the highest average rating (μ = 4.4, SD = 1.1) (Additional file 2: Tables 5, 6, 7 and 8). On the appropriateness scale, each of the items had a similar average rating, approximately (μ = 3.8, SD = 1.2). On the feasibility scale, the “TennCare MTM Pilot seems easy to implement into our practice” had the lowest average rating (μ = 3.0, SD = 1.4) and the “The TennCare MTM Pilot seems possible” had the highest average rating (μ = 4.0, SD = 1.1).

**Table 4:** Individual item Scores for Scales for Feasibility, Acceptability, and Appropriateness of the TennCare MTM Pilot Program

Scale itemsa	Mean (SD)	Range
Acceptability of intervention measure (AIM)^a^		
(AIM 1) I approve of the TennCare MTM Pilot	4.39 (1.14)	1–5
(AIM 2) The TennCare MTM Pilot is appealing to me	4.00 (1.19)	1–5
(AIM 3) I like the TennCare MTM Pilot	3.72 (1.23)	1–5
(AIM 4) I welcome the TennCare MTM Pilot as part of my practice	4.11 (1.28)	1–5
Intervention appropriateness measure (IAM)		
(IAM 1) The TennCare MTM Pilot seems fitting for my organization	3.83 (1.25)	1–5
(IAM 2) The TennCare MTM Pilot seems suitable for my organization	3.83 (1.15)	1–5
(IAM 3) The TennCare MTM Pilot seems applicable for my organization.	3.83 (1.24)	1–5
(IAM 4) The TennCare MTM Pilot seems like a good match for my organization	3.83 (1.29)	1–5
Feasibility of intervention measure (FIM)		
(FIM 1) The TennCare MTM Pilot seems implementable	3.89 (1.18)	1–5
(FIM 2) The TennCare MTM Pilot seems possible	4.00 (1.08)	1–5
(FIM 3) The TennCareMTM Pilot seems doable	3.83 (1.15)	1–5
(FIM 4) The TennCare MTM Pilot seems easy to implement into our practice	3.00 (1.37)	1–5

^a^ Each item was measured using a five point Likert scale

#### Contextual inquiry

Key stakeholders were primarily providers (physicians and pharmacists), MCOs, and Medicaid opinion leaders, including either organizational leadership or the primary MTM-providing pharmacist. Interviews were conducted at the informants place of employment. Saturation was reached with eight interviews. Interviews averaged 28 min in length.

### Process

#### Organizational-level

The MTM Pilot program implementation process varied widely across sites, was generally informal, and reactive to problems only after they arose (e.g., communication with MCOs, delays in credentialing). Planning happened in small, separate teams typically connected together by a champion. Contracting, credentialing, and other administrative plans were developed between the champion and organizational leadership. Day-to-day operational implementation typically occurred between champion and support staff (e.g., nurse or pharmacy technician). Champions for the program included the primary MTM-providing pharmacist and occasionally a pharmacist-extender (administrator or pharmacy technician). Formally appointed internal implementation leaders included the primary MTM-providing pharmacist, who generally was also the MTM champion. Other participants included medical office administrators, pharmacy technicians, medical assistants, physicians, nurses, and pharmacists.

*“We don’t really have anything I would say is concrete [to plan for implementation], but since I’m blessed with the nurse being three doors down from me, we’re actually in constant conversation about how to get started” (Informant 3, pharmacist)*

#### Pharmacist-level

Engagement and execution varied widely across sites during initial implementation as time was available and typically only after competing priorities were addressed first. Given small, informal implementation teams of 1–2 individuals per organization, reflecting and evaluating was limited mostly to self-reflection and direct feedback to TennCare; however, there were some exceptions to this. The planning phase was the terminal phase for several sites who noted that reimbursement fee schedules, duplicate documentation of care notes in both the care coordination tool (TennCare) and within the EHR (PCMH or THL), onboarding requirements (e.g. credentialing, training, MCO administrative requirements), and face-to-face visit requirements made the MTM pilot not compatible with existing organizational structure, workload, and workflow.

*“We went through the training and then talked to the boss about all the things that I’m going to have to do. And this is how much they are going to pay us. And again, the payment was kind of a kicker. Because of other sources [of MTM] like [the Medicare MTM program] where they’re paying [more] to do a comprehensive medication review with less paperwork and less time. It just wasn’t conducive to us right now. So, we then decided not to participate in the program. We did get our NPI. We went through all the steps to get here. But we just, at the end of the day, decided that it just wasn’t going to work for us at this time.” (Informant 4, pharmacist)*

#### Characteristics of individuals

Pharmacist participants across all settings noted that they were enthusiastic about the MTM pilot and felt it was important for Medicaid beneficiaries. Pharmacists who were already embedded in medical offices as providers of direct patient care before the pilot project (as opposed to those embedded in external, community pharmacy settings) noted that the program aligned with their organizational duties. Pharmacists practicing in more traditional community pharmacies generally noted it was their identification as a professional (and therefore a professional duty) to participate, rather than being directly related to organizational duties.

*“We’re the most accessible health care professional. I mean, literally [MTM is] what we’re trained for. No offense to nurses. We need them too. But this isn’t their ‘gig.’” (Informant 7, pharmacist)*

### Intervention characteristics

#### Organization-level

Adaptability was noted to be difficult and the program was seen as stringent in the requirement of having face-to-face encounters, pre-established payer goals for visits, and pre-determined eligible patient population (subset of entire state Medicaid population). Billing and use of required online MTM software platform (e.g. the care coordination tool) was noted to be difficult and often lagged, resulting in inefficiency. Similarly, complexity was noted as the required software was not embedded in current pharmacy dispensing or electronic health record software and so consequently pharmacists were required to perform duplicate documentation. Additionally, pharmacists expressed difficulty searching for eligible patients within the online care coordination tool.

*When I went and did a cursory review of [the online care coordination tool], the first five patients were not at my clinic. It took me six patients to even find one who was my clinic. I worry about the resources to actually find the patients. (Informant 1, pharmacist)*

As this was a pilot study, the program was designed to be rolled out in a way that allowed testing on a small scale and then changes to be made as needed, and so participants consequently reported a degree of trialability in the program. Open communication with TennCare further fostered trialability and subsequent feedback to researchers and the TennCare implementation team.

*“We were still at such a hurdle that last week my Chief Operations Officer … set up a meeting with [the TennCare project lead] and a couple of other key members at the TennCare program along with the leadership team from [my organization] just to see how they could finally break through these hurdles.” (Interview 3, pharmacist)*

#### Pharmacist-level

Pharmacists noted that MTM was an important intervention that addressed patient’s needs and had a strong evidence-base but was difficult to implement. They compared the relative advantage of the MTM pilot across several other clinical pharmacist services, such as Medicare’s MTM program and indicated significant advantages of the Medicare program over the Medicaid MTM pilot, including speed and simplicity of enrollment, usability of billing and care coordination software, and higher pharmacist fee schedules.

*“To me, personally, [payment] is not an issue. I know that to our organization, it may be. And that’s related entirely to the reimbursement structure of the MTM program. [The pharmacy organization] was very hesitant to actually partner in this pilot because the reimbursement and some of the things they would have to do to be able to be part of the [care coordination tool] was very concerning to them. So down the road, I could see that creating barriers that I might be able to foresee right now.” (Informant 3)*

### Inner setting

#### Organization-level

MTM aligned well with their organizational culture and tension for change. However, participants cited challenges with internal communication, accessing knowledge about the intervention, integrating the intervention into workflow, and fit between the intervention and the structural characteristics of the organization.

In terms of organizational culture, members of the PCMH/THL were receptive of pharmacist assistance and their incorporation into the care team. Across settings, the goal of the MTM project was noted to align with the mission of PCMH/THL or community pharmacies and the professional duties of the prescribers, pharmacists, and administrative staff. However, there was confusion due to differences between payer and provider goals, as pharmacists articulated their desire to serve their entire patient population, whereas the pilot included only those patients with certain disease states and risk scores.

In terms of organizational climate, organizational leaders had not created systems for recognizing or rewarding staff effort for implementation. Instead, motivation for participation was mainly driven by internal incentives (i.e. professional satisfaction and commitment to organization) and there were not clear expectations set from leadership regarding participation in the MTM pilot. Communicating the priority of the pilot amongst competing priorities within the organization was also noted to be a challenge.

There were also challenges with internal networks and communication. For example, there was difficulty establishing communication between medical schedulers and pharmacists to overlap physician visits with pharmacist MTM visits.

Workflow compatibility was varied across sites. Dedicated administrative or nursing staff to handle scheduling was a facilitator for sites with pharmacists embedded in the PCMH/THL; and lacking such personnel was conversely a barrier. For those pharmacists needing to both schedule and provide MTM services it was difficult to find a time for the patient to come in “between” regularly scheduled office visits.

*But what I’ve noticed is that when setting up the MTM appointments we have a high no-show rate... So, I think capturing patients that come into their [regularly scheduled physician] appointment or being able to set up the MTM appointment after [will help].” (Informant 5)*

Structural characteristics were noted to play a major role in implementation of the pilot. The lack of physically shared space between community pharmacists (those not already embedded in the PCMH/THL prior to the pilot) and medical offices was a barrier. In medical offices where pharmacists were already embedded, structure facilitated implementation more than for those located in community pharmacies. Community pharmacists’ frequent encounters with patients as part of usual prescription dispensing functions was a facilitator to conducting MTM “on the fly” while the patient was at the pharmacy, however.

“*… seeing the patients directly in the clinic it makes it easier to implement. Because we’re already doing something similar with our non-TennCare patients and so the providers are already used to us spending time with the patients one on one, doing medication reconciliation, and coming up with plans that we’re recommending to them. And so really the clinic structure is already there, it’s more just the documentation that’s different than what we’re already doing.”* (*Informant 5, Pharmacist*)

*“So the only thing that’s a little bit of a struggle for us … is we don’t have a consultation space, so to say. We don’t have a private, quiet space. So we would have to be able to work with the facility to create one for us for some of the face to face.” (Informant 3, Pharmacist)*

## Discussion

This study successfully demonstrated how an early implementation contextual analysis within a hybrid type 2 study design can be leveraged to identify and address initial barriers to the implementation [30, 31]. The critical value of such an assessment when implementing an evidence-based practice model in settings new to the evidence-based practice was highlighted by the dramatic increase in adoption after programmatic changes were made based that data. Moreover, the study emphasized the importance of rapidly assessing and disseminating contextual factors to program stakeholder so that implementation strategies may be adapted during the early phases of implementation.

Based on the real-time reporting of rapid analysis findings for early implementation barriers and facilitators, TennCare implemented several key changes to their implementation strategy in the last quarter of 2019 and first half of 2020 to address the decline in MTM claims that occurred in the summer of 2019. These included:

1. Promoting network weaving to overcome lacking stakeholder interrelationships: Conference call between North Carolina pharmacists experienced in Medicaid MTM implementation and Tennessee MTM pharmacists (Quarter 4, 2019)
2. Provide ongoing consultation to address technical barriers during implementation: Expert MTM implementation consultation (by University expert faculty) (Quarter 4, 2019)
3. Use Train the Trainer Strategies to expand the reach of program training (Quarter 4, 2019)
4. Pharmacist fee schedule increases to overcome financial barriers including overall cost of program implementation (Quarter 1, 2020)
5. A restructure strategy to decrease time to “go live” via a re-sequencing of MTM software training concurrent with credentialing process (rather than afterward) to reduce program complexity (Quarter 1, 2020)
6. Waiver of need for initial face-to-face visit to improve program compatibility (Quarter 1, 2020)
7. Duplicate documentation workaround to reduce program complexity and improve compatibility with existing workflows (Quarter 2, 2020),
8. Eligibility expansion to reduce program complexity (Quarter 3, 2020),

Program adoption, as measured by total number of MTM claims, increased during and after these implementation strategy changes (Fig. 2).

The primary goal of the study was to describe implementation outcomes and contextual determinants of a Medicaid MTM pilot program during the initial phase of implementation. To our knowledge this is the first study to assess the feasibility, acceptability, and appropriateness of an implementation strategy to support MTM delivery. Overall, the study found that key stakeholders are supportive of the MTM program, but significant challenges with implementation persisted despite a well-evidenced and multi-faceted implementation strategy to reduce barriers. For instance, in a similar MTM pilot project in North Carolina was successfully launched despite no pharmacist remuneration during the first year. In contrast, a significant barrier for Tennessee pharmacists was pharmacist remuneration – a barrier identified during contextual inquiry. We believe this particular finding is related to recent declining third party reimbursement for prescription drugs over the past few years resulting in increased financial strain for community pharmacies [32, 33].

While participants reported a moderately high-level of feasibility in the surveys, the qualitative data suggested that there were notable barriers that may have affected feasibility. For example, double documentation was cited consistently throughout interviews as a key implementation barrier. Although web-based MTM software systems allow for MTM program patient referral, documentation, and care coordination, the lack of interfacing with the software systems already implemented at the PCHM/THL or pharmacy represent a major barrier to implementation, especially as it relates to the extra time required for duplicate documentation [12]. Therefore, to increase feasibility, strategies, such as simplifying documentation requirements or providing enhanced education on efficient documentation, may be needed. Additionally, intervention complexity was frequently mentioned as a barrier, which is consistent with prior studies [34]. MTM, for some pharmacies, may represent a drastic shift from a prescription dispensing model to a more patient-centered workflow [34], requiring change at multiple levels (e.g., job roles, IT systems, workflow) [35, 36]. Given that pharmacies will likely vary in their preparedness for MTM, tailored implementation strategies may be needed to reduce barriers [14].

Despite noted barriers, stakeholders indicated a high level of support for the intervention. Most participants felt that the program aligned with their organization’s mission and the pharmacy profession. One reason for this may be due to the long-standing history of MTM services. Pharmacists have been providing MTM services since the 1990s [37, 38] and on a nationwide scale since the introduction of Medicare Part D in 2006 [39]. Given the familiarity of the profession with MTM, overall positive perceptions of the program are not surprising. The recent mandate of a doctoral-level pharmacy degree emphasizing clinical therapeutics and patient care have likely further influenced pharmacist perceptions of MTM, despite its adding to pharmacist workload and disrupting workflow.

Prior studies have suggested that partnerships between pharmacists, other healthcare providers, and social service agencies are a key facilitator of MTM programs [11]. The TennCare MTM Pilot used CPAs as an implementation strategy to facilitate better network ties across providers and pharmacists. However, partnership building was still cited as an early barrier to entry in the program among study participants. This suggests that implementation strategies may be needed to support partnership building and care coordination as a means to scale the program. Prior studies also suggest that payment models are critical to support MTM programs. While inclusion of a payment mechanism for pharmacist services was initially a facilitator in the TennCare MTM Pilot program, legislative restrictions led to a change from a value-based to a fee for service (FFS) model. Consequently, the FFS payment became a barrier as it was at a lower fee schedule than other reimbursable MTM programs.

Of note, there was no mention of external change agents or peer pressure during the key informant interviews, despite this having been shown to be a key driver for MTM implementation in prior studies [10, 40]. To address this gap, in the last quarter of 2019, a University-facilitated conference call was held to foster external collaboration with North Carolina pharmacists due to prior successful implementation of a Medicaid MTM program and similarities in practice region (Southeast United States). Anecdotal feedback was overwhelmingly positive and may represent another opportunity for future implementation strategies. Perhaps one of the reasons such external facilitation can prove useful for MTM implementation is learning of its successful implementation elsewhere given the general lack of compatibility between existing pharmacist workflows, especially those primarily of medication distribution, and clinical service delivery. Facilitated adaptation has been shown beneficial in these situations [14].

Across all settings, the MTM implementation plan was informal, iterative, and mainly reactive in nature. A review of the MTM implementation literature also reveals little formal implementation planning at the organizational level [36]. The use of a formal planning process, including models to guide implementation, has been shown beneficial in the implementation of new clinical initiatives in other professions [41, 42]. Although most sites did not have a formal plan for implementation, many sites did appoint a champion to help spearhead the intervention. As was true in previously reported MTM project implementation studies, our study found that engaging MTM champions was a clearly articulated driver of implementation even in the face of significant barriers. This is likely due to the large, intrinsic motivation to show the value of the pharmacist to patients and payers as pharmacists are not recognized as providers and reimbursement for patient care services remains largely elusive [36].

As a result of this early stage assessment, TennCare has made several programmatic changes. As of the first half 2020, the following changes are now in place: pharmacist reimbursement rate increases after a nationwide review of pharmacist MTM fee schedules, re-sequencing of care coordination tool training during credentialing process to increase the speed of program enrollment, duplicate documentation workaround using existing software limitations, and an eligibility expansion. TennCare is also investigating the feasibility of removing the requirement that the first visit with patients be face-to-face facilitate patient scheduling and reduce patient “no-show” appointment rates.

This study had several limitations. These included a small sample size (15 survey participants, 9 interview participants), which limits the generalizability of our findings. Recruiting study participants was difficult, likely because of challenges encountered with implementation. It is possible that participants in our study had more positive perceptions of implementation whereas non-responders, who really struggled with implementation of the TennCare MTM Pilot, were less likely to participate. This study also collected perceptions regarding implementation from the innovation users and did not capture perceptions from the purveyor—TennCare staff who helped support implementation of this pilot [22]. Future research should assess perceptions about implementation from the purveyors of MTM, who play a critical role in disseminating MTM and supporting pharmacists with implementation. Furthermore, the use of the eight programmatic changes to this MTM program listed above as an implementation strategy package should be explored in an experimental design against either a control, or as a comparative effectiveness study evaluating several implementation strategies.

## Conclusion

The early stage implementation of a multi-faceted implementation strategy to support delivery of Tennessee Medicaid’s MTM program was found to be well accepted and appropriate across multiple stakeholders including providers, administrators, and pharmacists. However, as the early stage of implementation progressed, barriers related to relative priority, characteristics of the intervention (e.g., complexity), and workflow impeded adoption. Programmatic changes to the MTM Pilot based on early stage contextual analysis and implementation outcomes had a positive impact on adoption.

## Supplementary Information

**Additional file 1.**

**Additional file 2.**

## Data Availability

The dataset (including individual transcripts) is not publicly available due to a confidentiality agreement between the research institution and TennCare.

## Abbreviations

CFIR: Consolidated framework for implementation research
MTM: Medication therapy management
CMS: Centers for Medicare and Medicaid Services
PCT: Primary Care Transformation
PCMH: Patient-centered Medical Home
CPA: Collaborative Practice Agreement
MCO: Managed Care Organization
CPT: Current Procedural Terminology
FIM: Feasibility Intervention Measure
AIM: Acceptability of Intervention Measure
IAM: Intervention Appropriateness Measure
COREQ: Consolidated Criteria for Reporting Qualitative
THL: Tennessee Health Link
