# Supplementary material for: The Tennessee Medicaid medication therapy management program: early stage contextual factors and implementation outcomes
Source: BMC Health Serv Res. 2021 Nov 2;21:1189. doi: 10.1186/s12913-021-07193-7 (PMC8561881; doi:10.1186/s12913-021-07193-7)
Supplement: Supplementary file 1 — Additional file 1. [file 12913_2021_7193_MOESM1_ESM.docx]

**Additional File 1**

**Table 5. Perceptions about acceptability, appropriateness, and feasibility**

| **Scale** | **Mean (SD)** | **Range** |
| --- | --- | --- |
| Acceptability of intervention measure (AIM), 4 items | 16.13 (5.01) | 4-20 |
| Intervention appropriateness measure (IAM), 4 items | 15.47 (5.25) | 4-20 |
| Feasibility of intervention measure (FIM), 4 items | 14.47 (4.63) | 4-20 |

Total observations: 15

**Table 6. Internal consistency of scales** (N=15)

| **Scale** | **Cronbach’s alpha (** α) |
| --- | --- |
| Acceptability of intervention measure (AIM), 4 items | 0.959 |
| Intervention appropriateness measure (IAM), 4 items | 0.985 |
| Feasibility of intervention measure (FIM), 4 items | 0.905 |

**Table 7. Correlation matrix for scales**

| **Scale items** | **Items** | | | |
| --- | --- | --- | --- | --- |
|  | 1 | 2 | 3 | 4 |
| ***Acceptability of intervention measure (AIM)*** | | | | |
| (AIM 1) I approve of the TennCare MTM Pilot | 1.000 |  |  |  |
| (AIM 2) The TennCare MTM Pilot is appealing to me | 0.865 | 1.000 |  |  |
| (AIM 3) I like the TennCare MTM Pilot | 0.709 | 0.887 | 1.000 |  |
| (AIM 4) I welcome the TennCare MTM Pilot as part of my practice | 0.934 | 0.930 | 0.808 | 1.000 |
| ***Intervention appropriateness measure (IAM)*** | | | | |
| (IAM 1) The TennCare MTM Pilot seems fitting for my organization | 1.000 |  |  |  |
| (IAM 2) The TennCare MTM Pilot seems suitable for my organization | 0.962 | 1.000 |  |  |
| (IAM 3) The TennCare MTM Pilot seems applicable for my organization. | 0.925 | 0.962 | 1.000 |  |
| (IAM 4) The TennCare MTM Pilot seems like a good match for my organization | 0.928 | 0.928 | 0.964 | 1.000 |
| ***Feasibility of intervention measure (FIM)*** | | | | |
| (FIM 1) The TennCare MTM Pilot seems implementable | 1.000 |  |  |  |
| (FIM 2) The TennCare MTM Pilot seems possible | 0.917 | 1.000 |  |  |
| (FIM 3) The TennCareMTM Pilot seems doable | 0.980 | 0.896 | 1.000 |  |
| (FIM 4) The TennCare MTM Pilot seems easy to implement into our practice | 0.544 | 0.514 | 0.522 | 1.000 |

**Table 8. Factor loadings for scales**

| **Scale items** | **Factors** | | | |
| --- | --- | --- | --- | --- |
|  | 1 | 2 | 3 | 4 |
| ***Acceptability of intervention measure (AIM)*** | | | | |
| (AIM 1) I approve of the TennCare MTM Pilot | *0.492* | -0.594 | **0.502** | 0.391 |
| (AIM 2) The TennCare MTM Pilot is appealing to me | *0.516* | 0.140 | -0.704 | 0.468 |
| (AIM 3) I like the TennCare MTM Pilot | *0.476* | **0.747** | 0.461 | -0.056 |
| (AIM 4) I welcome the TennCare MTM Pilot as part of my practice | *0.515* | -0.264 | -0.200 | -0.791 |
| ***Intervention appropriateness measure (IAM)*** | | | | |
| (IAM 1) The TennCare MTM Pilot seems fitting for my organization | *0.497* | **0.620** | 0.479 | 0.374 |
| (IAM 2) The TennCare MTM Pilot seems suitable for my organization | *0.502* | 0.356 | -0.523 | -0.590 |
| (IAM 3) The TennCare MTM Pilot seems applicable for my organization. | *0.502* | -0.403 | -0.472 | **0.603** |
| (IAM 4) The TennCare MTM Pilot seems like a good match for my organization | *0.498* | -0.572 | **0.525** | -0.387 |
| ***Feasibility of intervention measure (FIM)*** | | | | |
| (FIM 1) The TennCare MTM Pilot seems implementable | *0.543* | -0.205 | -0.310 | -0.754 |
| (FIM 2) The TennCare MTM Pilot seems possible | *0.525* | -0.220 | 0.816 | 0.102 |
| (FIM 3) The TennCareMTM Pilot seems doable | *0.536* | -0.230 | -0.487 | **0.649** |
| (FIM 4) The TennCare MTM Pilot seems easy to implement into our practice | *0.378* | **0.926** | 0.004 | 0.019 |

Note: Factor loadings in boldface indicate double loading on two or more factors. Factor loadings in italics indicate the factor on which the item was placed.
