# Supplementary material for: The Tennessee Medicaid medication therapy management program: early stage contextual factors and implementation outcomes
Source: BMC Health Serv Res. 2021 Nov 2;21:1189. doi: 10.1186/s12913-021-07193-7 (PMC8561881; doi:10.1186/s12913-021-07193-7)
Supplement: Supplementary file 2 — Additional file 2. [file 12913_2021_7193_MOESM2_ESM.docx]

Additional File 1

Demographics

1. What is your role within the organization?

How long have you been in this role?

How long have you been with the organization?

Besides yourself, who else would you recommend us interviewing? What is the best way of getting in contact with them?

Inner

Setting

*Structural*

*Characteristics*

1. How will the infrastructure of your organization (social architecture, age, maturity, size, or physical layout) affect the implementation of the TennCare MTM Pilot?

- How will the infrastructure facilitate/hinder implementation of the TennCare MTM Pilot?
- How will you work around structural challenges?

# *Culture*

3. How will your organization's culture (general beliefs, values, assumptions that people embrace) affect the implementation of the TennCare MTM Pilot.

# Implementation Climate

## Tension for Change

4. To what extent is there a strong need for the TennCare MTM Pilot?

o How do you perceive others at your organization seeing a need for the MTM Pilot?

## Compatibility

5. How well does the TennCare MTM Pilot fit with existing work processes and practices in your setting?

- What kinds of changes or alternations, if any, do you think you will need to make the TennCare Pilot so it will work effectively in your setting?
- What components, if any, should not be altered? What about the pilot do you think works well and shouldn’t be changed?

## Relative Priority

6. To what extent might the implementation take a backseat to other high-priority initiatives going on now?

- How important do you think it is to implement the TennCare MTM Pilot compared to the other priorities?
- How important is it to others, such as your coworkers or leaders, to implement the TennCare MTM Pilot compared to the other priorities?

### Organizational Incentives & Rewards

7. What kinds of incentives, if any, are there to help ensure that the implementation of the TennCare MTM Pilot is successful?

o What is your motivation for wanting to help ensure the implementation is successful?

Readiness for Implementation

### Leadership Engagement

8. What level of involvement has leadership at your organization had so far with the TennCare MTM Pilot?

- Who are these leaders? How do attitudes of different leaders vary regarding the TennCare MTM Pilot?
- Give an example of the support they have given you.

### Available Resources

1. Do you expect to have sufficient resources to implement and administer the TennCare MTM Pilot?
   - If YES x What resources are you counting on? Are there any other resources that you received, or would have liked to receive?

What resources will be easy to procure?

If NO - What resources will not be available?

1. Which staff members within your organization will be involved with implementing the TennCare MTM pilot?

Characteristics

of

Individuals

Knowledge

&

Beliefs

about

the

Intervention

1. How have the staff in your organization responded to the TennCare MTM pilot?
   - Have any of the staff been unwilling or resistant to working on the TennCare MTM pilot?
2. How do you feel about the TennCare MTM Pilot being used in your setting?
   - How do you feel about the plan to implement the TennCare MTM Pilot in your setting? o Do you have any feelings of anticipation? Stress? Enthusiasm? Why or why not?

Process

# Planning

12. What have you done (or what do you plan to do) to get a plan in place to implement the TennCare MTM Pilot?

# Formally Appointed Internal Implementation Leaders (Champions)

13. Who will lead implementation of the TennCare MTM Pilot within your organization?

- How did/will this person come into this role? Appointed? Volunteered? Voluntold?
- What attributes or qualities does this person have that makes them an effective leader of this implementation? What attributes or qualities does this person lack?
- To what extent does this person have sufficient authority to do what is necessary to implement the TennCare MTM Pilot?
